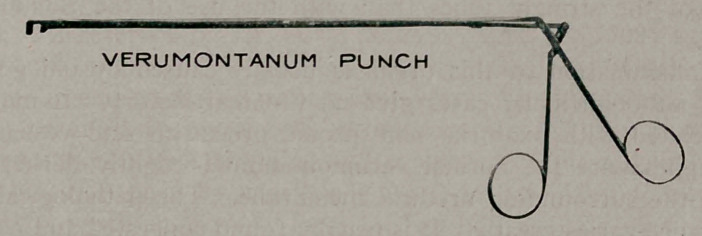# Inflammation of the Verumontanum and Posterior Urethra with Presentation of an Instrument

**Published:** 1912-06

**Authors:** James A. Gardner

**Affiliations:** Attending Genitourinary Surgeon, German Hospital; Consulting Urologist, Columbus Hospital and St. Francis Asylum. Buffalo, N. Y.


### Inflammation of the Verumontanum and Posterior Urethra with Presentation of an Instrument.

By JAMES A. GARDNER, M. D.
Attending Genitourinary Surgeon. German Hospital; Consulting Urologist, Columbus Hospital and St. Francis Asylum.
Buffalo, N. Y.

MORE attention has been paid in recent years than formerly to the pathological conditions found in the posterior urethra. This increased interest has been brought about primarily by the improvement in instruments and technic and the urethra is now examined and treated with the same accuracy as the nose and throat. One of the interesting and important parts of the posterior urethra is the verumontanum, which, before the introduction of the improved instruments, apparently had been overlooked. The anatomies do not agree upon the location or size of the verumontanum and the descriptions are most hazy. It contains, as you know, the openings of the ejaculatory ducts and arises from the floor of the posterior urethra not unlike a nose, but as no two noses are alike so no two normal verumontani are the same. My attention was first called to the importance of this organ by Dr. Young, who was achieving such satisfactory results in certain classes of cases in so short a time that his success seemed hardly possible when one considered the length of time we had worked over similar cases, often with but little improvement.

To Swinburne should be given the credit of recognizing the importance of this organ and the endoscope which bears his name, has been of great aid in its treatment. The organ is of much more importance than has been realized. It is richly endowed with nerves and can give rise to motor and sensory symptoms of both the genital and urinary tract. The verumontanum is best seen, I believe, through the Swinburne tube. After passing the tube completely into the bladder and withdrawing it slowly half an inch or so, the organ appears in the window. An aspirator will aid in withdrawing the small amount of urine that may obscure the view. I think the Swinburne tube with a light near the window gives not only the best view but also is much more comfortable for the patient during its insertion. Dr. Young prefers a straight tube or Swinburne tube with reflected light because he has more room to work in the endoscope. However, I have not been able to get as clear a view with tubes having reflected light as with those having the light in the tube, and my patients have complained of more pain during the introduction of the straight tubes than with the use of the Swinburne tube.

Inflammation of this organ is usually caused by old gonorrhea although many cases give no venereal history. It may be associated with oxaluria, non-specific prostatitis and vesiculitis. In appearance the normal verumontanum is sightly darker red than the surrounding urethral membrane. The pathological appearance varies greatly. It is usually found congested and hyperemic and bleeds on touch. It looks not unlike the wattles of a turkey. Many times there is a real hypertrophy. It may bulge on either side of the urethra and in some instances seems almost to fill it. An ulcer is often found upon the surface. Many of the symptoms complained of cannot be differentiated from inflammation of the prostate and vesicles, as has been shown by Hawkins.

When we consider that nine out of ten cases of anterior gonorrhea become posterior and that in almost the same ratio the prostate and seminal vesicles become infected, it can easily be seen why the verumontanum is also included in the inflammatory area. The wonder is how it escapes. After careful and diligent treatment of the prostate and vesicles by massage, it seems impossible often to completely clear up the urine. There will still remain a few shreds and pus cells. In the past we were taught to pass sounds and make deep instillations of 1 or 2 per cent. nitrate of silver. This treatment was effective sometimes but often apparently useless. We were not hitting the nail on the head but were applying a solution over an inch or more of the urethra when an eighth only was affected. The patient may give a history of burning sensation during urination or of sharp pains in the perineum. Frequent desire to urinate with tenesmus may exist and a few drops of blood may follow micturition. The pain is oftentimes referred to the rectum, hypogastrium or shoots down the legs. As in other conditions in the posterior urethra the pain frequently is complained of in the glans. Swinburne states that these pains generally are independent of the act of urination or may come at the end of urination. A neurotic condition is usually present. The irritation has caused nocturnal emissions and some patients complain of diurnal. Premature or delayed ejaculations and almost complete impotence are found. This condition is the cause of neurasthenia in a great many cases which cause is often overlooked. Because the patient gives no history referable to the genitourinary tract and his urine upon examination does not disclose much of anything, that part of his anatomy is excluded.

In illustration of some of the phases of these conditions I would like to cite a few cases.

L. M. referred by Dr. G. F. Reusch, April 13, 1909; age 22, telegraph operator. No venereal history, complains of pain over pubes, lumbar backache lasting four years; was operated on two years previous for varicocele, no improvement. Urine negative, verumontanum hypertrophied and congested. Touched with Ag. No. 3 stick; April 24, curretted; April 29 and May 4, treated; May 11, much improved; June 15, has had no pain over pubes or backache since last visit; July 15, has had no trouble since last visit; cured.

E. L. first seen January 7, 1907; street car conductor; age 31; gives history of five attacks of gonorrhea since he was fifteen years old. For past three years has noticed morning drop, smarting in posterior urethra and frequent urination and pain over pubes. Examination showed boggy prostate and engorged vesicles. Patient is marked neurasthenic. He was massaged, treated with instillation of nitrate silver, dilated for four months but the burning sensation and morning drop though improved still persisted; no gonococci present. I did not see patient again until June 23, 1909; he stated he had been under care of a number of physicians during the two years interval; still complained of smarting sensation at time of urination, pain over pubes; this is not affected by the quantity of urine in bladder. Examination showed verumontanum hypertrophied and congested, bleeds on touch. Amputated and touched with stick nitrate. June 28, has not noticed pain until this A. M.; August 9, has had four treatments since June 28, does not have pain but rarely; August 28, no pain since last visit; October 2, no pain since August.

Dr. J. D. referred by Dr. Stockton, October 20, 1909; age 41; neurotic. History of gonorrhea at 23 lasting for long time; had either exacerbation or new attack at thirty-three lasting four weeks; complains of pain in perineum and in left hypogastric region. Examination showed right seminal vesicle tender, verumontanum congested. Treated October 20, 28 and November 4. Cured.

F. W. V. referred by Dr. Walter A. Scott, of Niagara Falls, August 6, 1909; age 25, street car conductor; had gonorrhoea in 1906, lasting two years. During March began to have frequent painful urination; this continued and he was compelled to quit work the latter part of June. When I saw him with Dr. Scott on August 6, he could not hold his urine over an hour. Examination showed highly congested and hypertrophied verumontanum; was sent to bed for one week. Verumontanum treated August 11, 16 and 30; cured. Capacity 18 ounces, urinates about every four hours, does not get up at night.

P. D. referred by Dr. Charles Walrath, Ellicottville; age 58, farmer. Began to have pain at neck of bladder ten years ago; at first only noticed it when at rest, later it increased until he was conscious of it most of the time; had been under care of a number of physicians and been to various sanitariums. At one time was committed to State Hospital because physician thought he imagined he had pain. Saw him first October 26, 1911. Examination showed he had very sensitive right vesicle, small prostate, four ounces of residual urine and a highly inflamed hypertrophied verumontanum which bled on touch with cotton swab. Massage of vesicle and application of stick nitrate to verumontanum caused him to report November 4, without any residual urine and pain very much improved.

In the past upon passing a sound the patient did not complain of any discomfort upon the passage through the anterior urethra but upon entrance to the posterior urethra complained that the instrument caused him considerable pain. I make it a rule now when a patient complains of pain upon passage of an instrument through the posterior urethra to examine with an endoscope and am surprised to find how frequent the cause is a diseased verumontanum.

The treatment that has been found most efficacious is amputation of the large hypertrophies followed by application with the pure stick nitrate of silver. I have tried iodine, icthyol and various other drugs but without satisfaction.

I show an instrument which I call a punch, for want of a better name. You will notice that although the instrument is slender it is very strong because of its direct drive. A bite or two taken from the tumor will cause a marked decrease in size. In the majority of cases the application of the silver nitrate alone is sufficient. In other cases the currette will take the place of the punch. This class of cases has been of great interest to me. I have seen over 225 cases and each one seems to present something novel. When the condition is present in a nervous highstrung individual, it may readily give rise to a secondary neurotic tendency far out of proportion to the original existing cause.

403 FRANKLIN STREET.

## Figures and Tables

**Figure f1:**